# Kinetic Features of Degradation of R-Loops by RNase H1 from *Escherichia coli*

**DOI:** 10.3390/ijms252212263

**Published:** 2024-11-15

**Authors:** Aleksandra A. Kuznetsova, Iurii A. Kosarev, Nadezhda A. Timofeyeva, Darya S. Novopashina, Nikita A. Kuznetsov

**Affiliations:** 1 Institute of Chemical Biology and Fundamental Medicine, Siberian Branch of Russian Academy of Sciences, Novosibirsk 630090, Russia; y.kosarev@g.nsu.ru (I.A.K.); na_timof@niboch.nsc.ru (N.A.T.); danov@niboch.nsc.ru (D.S.N.); 2 Department of Natural Sciences, Novosibirsk State University, Novosibirsk 630090, Russia

**Keywords:** transcription, RNase H1, RNA polymerase, R-loop, enzyme activity, enzyme kinetics

## Abstract

R-loops can act as replication fork barriers, creating transcription–replication collisions and inducing replication stress by arresting DNA synthesis, thereby possibly causing aberrant processing and the formation of DNA strand breaks. RNase H1 (RH1) is one of the enzymes that participates in R-loop degradation by cleaving the RNA strand within a hybrid RNA–DNA duplex. In this study, the kinetic features of the interaction of RH1 from *Escherichia coli* with R-loops of various structures were investigated. It was found that the values of the dissociation constants *K*_d_ were minimal for complexes of RH1 with model R-loops containing a 10–11-nt RNA–DNA hybrid part, indicating effective binding. Analysis of the kinetics of RNA degradation in the R-loops by RH1 revealed that the rate-limiting step of the process was catalytic-complex formation. In the presence of RNA polymerase, the R-loops containing a ≤16-nt RNA–DNA hybrid part were efficiently protected from cleavage by RH1. In contrast, R-loops containing longer RNA–DNA hybrid parts, as a model of an abnormal transcription process, were not protected by RNA polymerase and were effectively digested by RH1.

## 1. Introduction

In modern terms, the bacterial nucleoid is a riboid representing a biomolecular condensate surrounded by another biomolecular condensate. This is established through liquid–liquid phase separation driven by differences in hydrophobicity and macromolecular mobility patterns between the two phases, and this phenomenon is supported only by a transcription zone and by intrinsically disorderly protein regions at a nucleoid–riboid interphase [1].

Transcribing RNA polymerases (RNAPs) in growing cells are the most numerous nucleoid-associated enzymes [2]. Modern super-resolution imaging of a GFP-labeled RNAP in *Escherichia coli* under different conditions of growth has clearly shown a distribution of the RNAP signal around the nucleoid periphery, enshrouding the DNA signal [3,4,5]. Recent single-molecule-resolution imaging of the RNAP distribution around a nucleoid confirmed that the transcribing RNAPs are prone to assemble at the nucleoid periphery, covering it with a network of clusters. At the same time, the “mobile” (empty) RNAPs explore the whole nucleoid volume [6,7]. DNA replication is carried out by replication machinery called the replisome [8], a multiprotein DNA replication apparatus [9]. Replication forks proceed in two directions (clockwise and counterclockwise) along a chromosome, thus replicating the genome at a rate of ~500–1000 bp per second. When replication forks are moving along a chromosome, they can collide with various obstructions, including the transcription apparatus, DNA-binding proteins, and different secondary structures of DNA or RNA. The collisions between replication and transcription machineries result in conflicts, and an inability to resolve these conflicts can result in genome instability, chromosomal rearrangements, and deletions [10,11].

Conflicts can occur at DNA lesions [12], as well as independently of pre-existing DNA damage [13], and replication is disrupted not only after head-on collisions with transcription machinery [14,15] but also during unidirectional collisions on a chromosome [16] or plasmid [17]. Little is known about what occurs with replication machinery at the sites of conflict; moreover, different types of conflict probably trigger dissimilar repair mechanisms [18,19,20,21,22]. The bacterial cell has a variety of strategies to avoid and resolve these conflicts and conserve genome integrity [11].

Depending on the orientation of a gene, replication machinery can encounter RNAPs either head-on or unidirectionally. The expression of genes that are encoded on the leading strand is coordinated with replication, whereas genes encoded on the lagging strand lie in the opposite orientation toward replication. A head-on or unidirectional conflict can disrupt replication in vivo, and auxiliary factors are involved in the resolution of replication–transcription conflicts [16,17].

As transcription proceeds, the nascent RNA is usually displaced from the DNA template strand within the transcription bubble. However, the high level of negative supercoiling directly behind the moving RNAP can promote DNA opening. As a result, the nascent RNA coming out of the RNA exit channel can reanneal to the DNA template strand, resulting in the formation of an RNA–DNA hybrid (i.e., an R-loop: the template strand is hybridized with RNA, thereby leaving the nontemplate strand DNA unpaired) [23]. Extensive R-loop formation was shown to occur during the transcription of plasmid DNA in the presence of DNA gyrase in vitro and in vivo [24,25,26,27]. Such R-loop formation on plasmid DNA has been linked to growth inhibition [28], gene expression defects [29,30,31], and unregulated replication [32,33] in *topA*-null mutants when RNase H1 (RH1, EC 3.1.26.4) is not overproduced. RH1 is an endonuclease that hydrolyzes the RNA strands in RNA–DNA hybrids [34]. RH1 participates in DNA replication and eliminating RNA oligonucleotides from Okazaki fragments on the lagging strand [35].

In the last decade, R-loops have attracted significant attention as important regulators of cellular processes and participants in phenomena such as DNA replication initiation in bacterial ColE1-type plasmids [36,37], immunoglobulin class switch recombination [38], the completion of mitochondrial-DNA replication [39,40], the facilitation of transcription termination [41], and the initiation of DNA double-strand break repair [42,43]. Nonetheless, extemporaneous R-loops can be an origin of genome instability. The RNA binding by ribosomes in bacteria and by proteins related to splicing, RNA transport, and RNA modification in eukaryotes normally prevents the formation of extemporaneous R-loops [44,45].

Although the direct relationship between R-loops and genomic stress has been difficult to establish, R-loops probably represent barriers for the replication fork, creating transcription–replication collisions [46,47,48] and inducing replication stress by arresting DNA synthesis [49,50], thus possibly causing aberrant processing and the formation of double-strand breaks [51,52]. DNA breaks can also occur in the single-stranded-DNA components of R-loops due to their exposure to reactive oxygen species and single-stranded-DNA nucleases, making R-loops susceptible to DNA damage [53]. Similarly, R-loops may cause transcription stress by blocking the advancement of RNAP, similar to how they interfere with replication. Transcription-coupled nucleotide excision repair, which normally removes bulky lesions from DNA that prevent transcription, may cleave the R-loops, thus producing DNA breaks and damage [52]. The best evidence that R-loops play a role in the induction of DNA damage comes from studies involving RH1: an enzyme that processes R-loops by cleaving the RNA strand of the RNA–DNA hybrid [54]. In vivo overexpression of RH1 in various systems, from *E. coli* to humans, partially suppresses diverse genome instability phenotypes [28,55,56], and this finding has helped to establish a relation between R-loops and DNA damage.

RH1 is used as a reporter for the detection of R-loops [57,58,59]. Nevertheless, there is no biochemical characterization of *E. coli* RH1’s interaction with R-loops in vitro. In the present study, we focused on the kinetic features of the interaction of RH1 with the R-loops of various structures. In addition, the degradation of an RNA primer was investigated in the case of an RNAP-bound R-loop.

## 2. Results

### 2.1. Design of R-Loops

During transcription, nascent RNA can hybridize with the DNA template strand, thereby leaving the nontemplate DNA strand single-stranded. The hybrid DNA–RNA duplex inside a transcription elongation complex is typically not longer than 10–11 bp, and its length is controlled by a special wedge-like structure of RNAP [60]. Table 1 contains the sequences of the model R-loops. R-loop 1 represents an 11 bp R-loop in a transcription elongation complex. R-loops 2 and 3 represent a possible RNA primer shift inside a transcription elongation complex. R-loops 4 and 5 represent an example of possible incorrect RNA primer elongation by RNAP resulting in the appearance of hanging noncomplementary nucleotides at the 3′ end.

**Figure 1:**
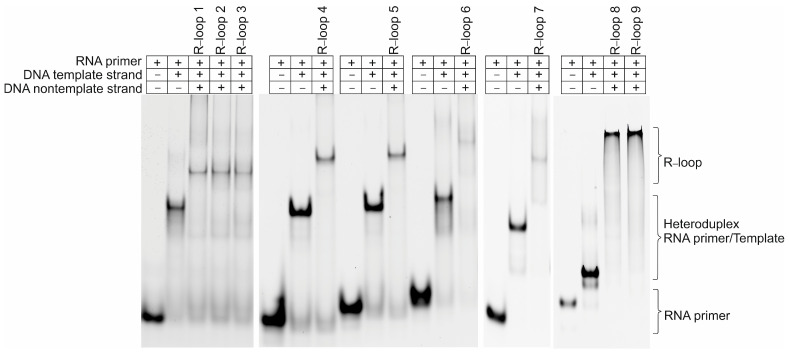
The EMSA of the R-loops. The concentration of the RNA primer and of the DNA template and nontemplate strands was 1 µM. The presence/absence of components in the mixture is indicated by +/– signs.

The nascent RNA is usually displaced from the DNA template strand within the transcription bubble during transcription. However, the high level of negative supercoiling directly behind the moving RNAP can promote DNA opening. As a result, the nascent RNA coming out of the RNA exit channel can reanneal to the DNA template strand [21]. To investigate the effect of the length of the RNA–DNA hybrid part, R-loops 6–8 were constructed. R-loop 9 contains a G-quadruplex in the DNA nontemplate strand. G-rich DNA strands with multiple repeats of guanine residues can promote the formation of secondary structures such as G-quadruplexes that can contribute to the R-loop’s stability. G-quadruplexes can regulate gene expression and other processes in bacteria [61,62], and it was of interest to assess the effect of a G-quadruplex on RH1’s action. The R-loops’ assembly was confirmed using EMSA (Figure 1). Successful formation of all the designed R-loops was observed under the reaction conditions.

The Gibbs free energy (ΔG) values for the DNA duplex with a bubble and heterodimer consisting of an RNA primer and template DNA chain were calculated using the IDT OligoAnalyzer Tool (https://eu.idtdna.com/calc/analyzer, accessed on 1 November 2024). For template/nontemplate DNA duplex formation, ΔG was –34.45 kcal/mol; for RNA primer/template DNA heterodimer complexes, ΔG decreased in the following order: R–loops 4 and 5 (–17.68 kcal/mol) > R–loops 1–3 (–19.64 kcal/mol) > R–loops 6–9 (–29.34, –35.79, –55.6, and –55.6 kcal/mol). This indicated that the designed R–loops were highly stable.

### 2.2. R-Loop-Binding Assays

**Figure 2:**
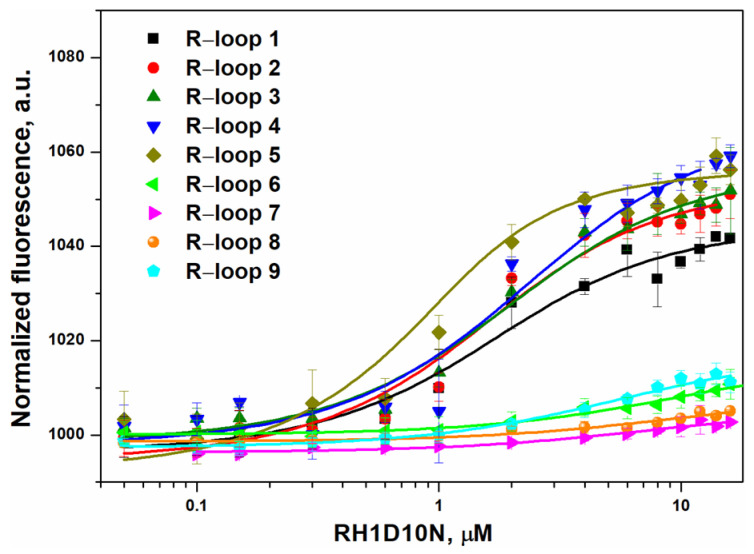
MST curves characterizing the interaction of RH1 D10N with R-loops.

A microscale thermophoresis (MST) analysis of the R-loops’ binding by RH1 was conducted using the catalytically inactive mutant RH1 D10N [63] to avoid RNA primer digestion during the measurement (Figure 2). It was shown that D10N amino acid substitution did not affect the *K*_m_ parameter but resulted in almost complete loss of activity, suggesting that it was involved in the catalytic activity rather than substrate binding [63]. The respective dissociation constants *K*_d_ were calculated and are summarized in Table 2.

A comparison of dissociation constants *K*_d_ revealed good binding of RH1 D10N to R-loops. For R-loops 1–4, the constants were not dependent on the R-loop structure. For R-loop 5, a 3.8-fold lower *K*_d_ was noted in comparison with that for R-loop 1, indicating stabler complex formation. An increase in the length of the RNA–DNA hybrid part (R-loops 6–9) resulted in a decrease in *K*_d_, implying less stable complex formation. Unfortunately, for R-loops 6–9, a saturating concentration of RH1D10N was not achieved. Therefore, the data fitting for the *K*_d_ values reflected the low limit estimation value.

### 2.3. RNase H Activity Assay

**Figure 3:**
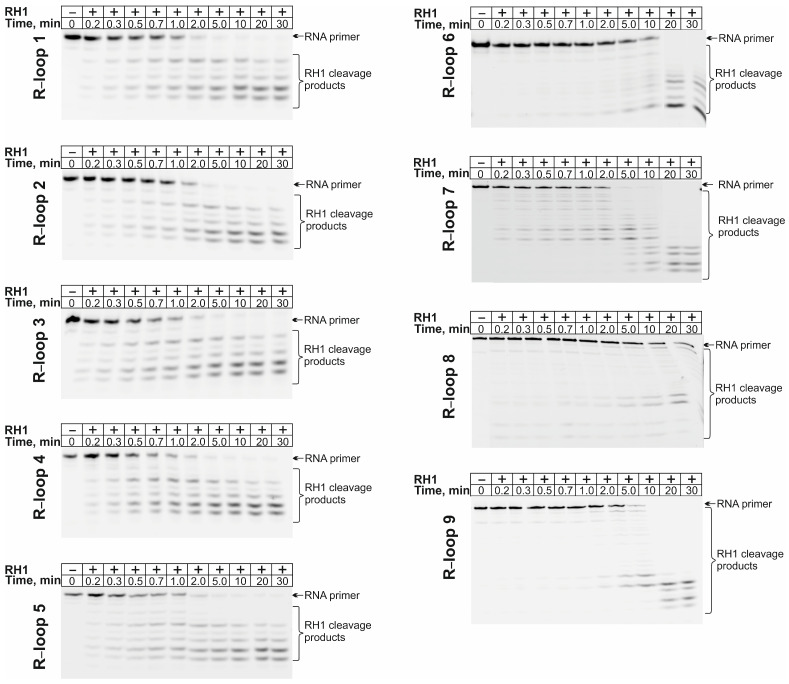
The RH1-driven cleavage of an RNA primer contained in the R-loops. The R-loops’ concentrations were 0.5 µM, and the RH1 concentration was 25 nM. Lanes show product accumulation for 0, 10, 20, 30, and 40 s and 1, 2, 5, 10, 20, and 30 min. The presence/absence of components in the mixture is indicated by +/– signs.

The kinetic analysis of the RH1-driven cleavage of the RNA primer contained in R-loops was performed next (Figure 3). For each R-loop, a series of kinetic curves was obtained in which the R-loop concentration was 0.5 μM and the enzyme concentration varied from 10 to 50 nM. The RNA primer cleavage by RH1 in R-loops 1–3 proceeded with the same efficacy. The presence of three hanging noncomplementary nucleotides in R-loop 5 at the 3′ end of the RNA primer led to a higher RNA primer cleavage rate. On the other hand, in R-loop 4 (with an RNA primer containing two hanging noncomplementary nucleotides at the 3′ end), the RNA primer cleavage took place with the same efficacy as for R-loop 1. The length of the RNA–DNA hybrid part (R-loops 6–8) diminished the cleavage rate, especially for R-loop 8. The most likely reason is a local increase in the number of “landing sites” for RH1 in the case of the long RNA–DNA hybrid part in R-loop 8 in comparison with R-loop 1. Notably, for R-loop 9 (with the same long RNA–DNA hybrid part as in R-loop 8 but with a G-quadruplex in the DNA nontemplate strand), the decrease in the cleavage rate was not so pronounced, maybe owing to a considerable deformation of the global R-loop structure.

**Figure 4:**
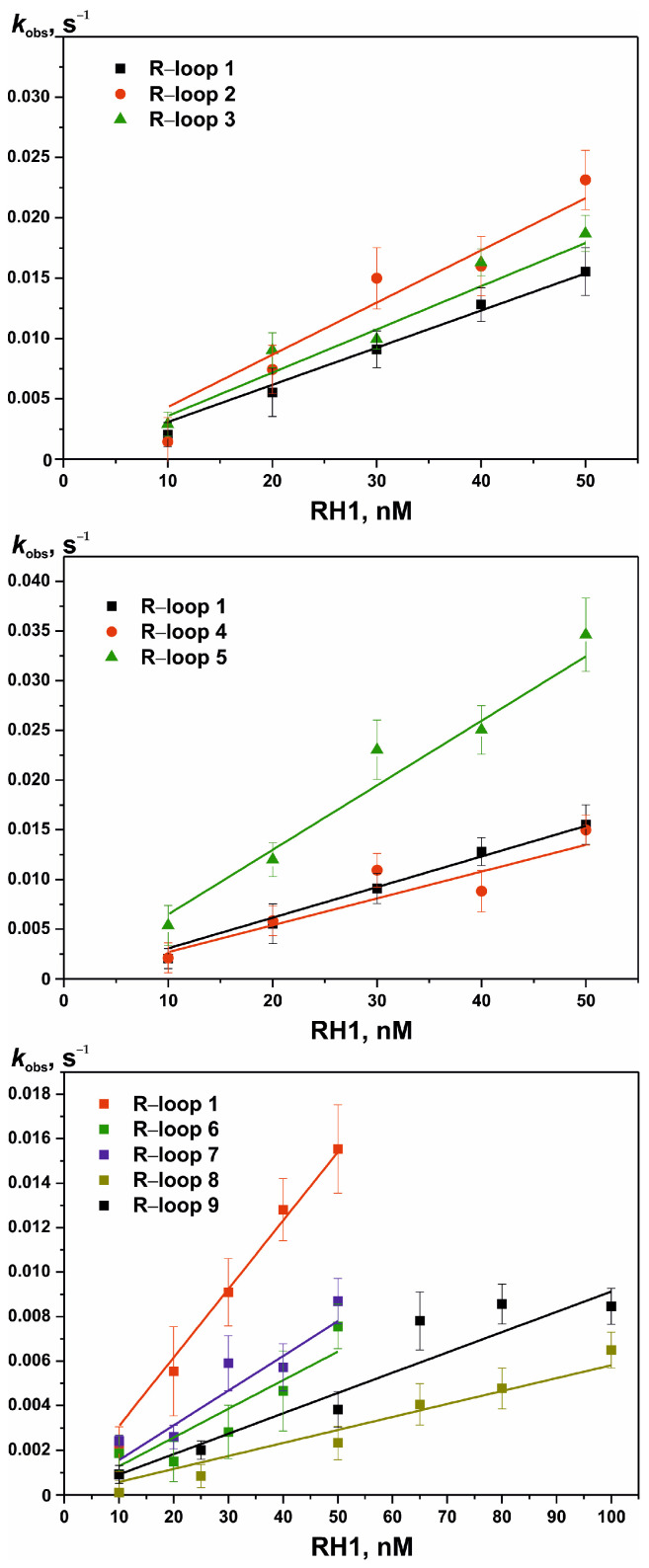
The dependence of the observed rate constant *k*_obs_ of the RNA primer cleavage on the RH1 concentration.

The kinetic curves of the RNA primer cleavage rate were well described by an exponential equation, which allowed us to determine the observed rate constant *k*_obs_. The dependences of the observed rate constants on the initial RH1 concentration were linear (Figure 4).

**Scheme 1:**
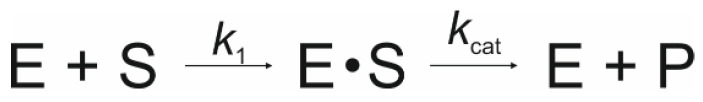
The kinetic scheme of the RH1-driven cleavage of an RNA primer contained in R-loops. E: RH1; S: an R-loop; E•S: The catalytic complex of the R-loop with the enzyme; P: a reaction product.

The linearity of the dependence indicated that the observed rate constant characterizes a bimolecular process and allows one to calculate the apparent rate constants of product formation using the equation *k*_obs_ = *k*_1_ × [RH1] (Table 2). In this case, the kinetic scheme of the enzymatic process can be described as Scheme 1, and the value of the rate constant of the catalytic reaction must conform to the condition *k*_cat_ >> S × *k*_1_. The *k*_cat_ parameter characterizes the cleavage of the phosphodiester bond, and it can be theorized that the conformation of the DNA–RNA hybrids does not significantly affect this kinetic parameter of the enzyme and that *k*_cat_ is the same for all R-loops.

Thus, the rate-limiting step of the R-loop degradation is catalytic-complex formation and depends on the rate constant *k*_1_. As one can see in Table 2, the *k*_1_ values for the formation of the catalytic complex of RH1 with R-loops are in good agreement with the *K*_d_ values obtained in the MST experiment: the extension of the RNA–DNA hybrid part (R-loops 6–9) resulted in a decline in *k*_1_.

### 2.4. RNase H Activity Assayactivity Assay in the Presence of the RNAP

**Figure 5:**
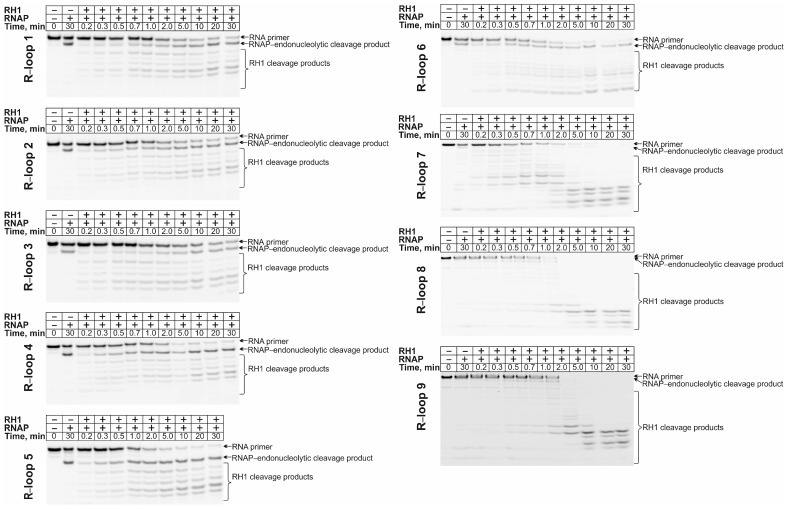
The RH1-driven cleavage of an RNA primer contained in the R-loops in the presence of the RNAP. The R-loops’ concentrations were 0.5 µM, the RNAP concentration was 1.0 µM, and the RH1 concentration was 25 nM. The lanes show product accumulation for 0, 10, 20, 30, and 40 s and 1, 2, 5, 10, 20, and 30 min. The presence/absence of components in the mixture is indicated by +/– signs.

To reconstruct the molecular interactions between the participants in a transcription–replication collision in vitro, the RH1-driven cleavage of an RNA primer contained in the R-loops in the presence of the RNAP was investigated next (Figure 5). For this purpose, a pre-formed [RNAP•R-loop] complex was assembled. The *K*_d_ of the RNAP with R-loop 1 as estimated with the MST procedure was 0.3 ± 0.1 µM. This enabled us to estimate the [RNAP•R-loop] complex concentration according to Equation (1) as 0.34 µM, which corresponds to ~68% R-loop binding.

(1) $$\left[ES\right]=0.5\times \left(\left(E_{0}+S_{0}+K_{d}\right)-\sqrt{{\left(E_{0}+S_{0}+K_{d}\right)}^{2}-4\times E_{0}\times S_{0}}\right),$$

where E_0_ is the initial concentration of the RNAP, S_0_ denotes the initial concentration of an R-loop, and *K*_d_ is the dissociation constant.

**Figure 6:**
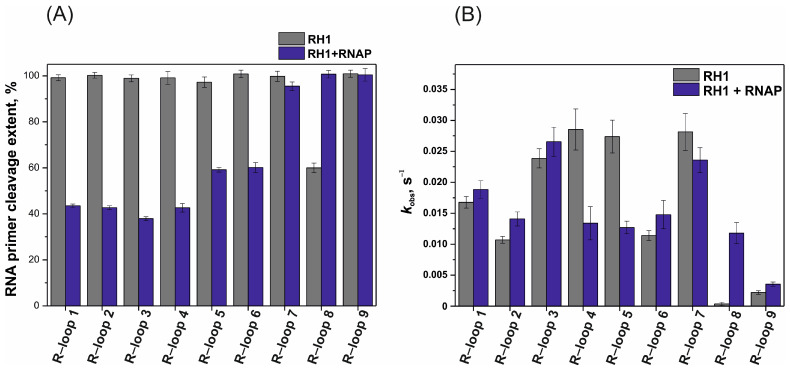
(**A**) The extent of the cleavage of an RNA primer by RH1 in the absence (gray) or presence (blue) of the RNAP. (**B**) Observed rate constant *k*_obs_ for RNA primer cleavage induced by RH1 in the absence (gray) or presence (blue) of the RNAP. The extent of the cleavage of an RNA primer was calculated at time point 30 min.

Because no NTPs were added to the reaction mixture, there was no advancement of the RNAP. Figure 5 shows the products of RNAP-induced endonucleolytic cleavage formed for each type of R-loop. The yield of the product of RNAP-induced endonucleolytic cleavage in the absence of RH1 varied from 10% to 45%; moreover, in the presence of RH1, a product of RNAP-induced endonucleolytic cleavage persisted for up to 30 min of reaction time with R-loops 1–6. This observation suggested that, after complex formation with R-loops 1–6, the RNAP does not dissociate, thus preventing the full degradation of an RNA primer (Figure 6A). On the other hand, for R-loops 7–9, such protection of an RNA primer by the RNAP was not observed. Furthermore, for R-loop 8, full degradation of the RNA primer by RH1 in the presence of the RNAP was observed, whereas in the absence of the RNAP, the RNA primer conversion was only 60% (Figure 6A).

The values of *k*_obs_ for the RNA primer cleavage were compared in the presence and absence of the RNAP (Figure 6B). As one can see, for R-loops 1–3, the presence of the RNAP was not reflected in *k*_obs_. It can be hypothesized that, in this case, RH1 interacted with a “free” R-loop unbound by the RNAP, thus resulting in a decrease in the overall extent of primer cleavage but not in the cleavage rate. For R-loops 4 and 5 (containing, respectively, two or three hanging noncomplementary nucleotides at the 3′ end of the RNA primer), a decline in the overall extent of primer cleavage was also documented. It should be pointed out that a 2-fold decrease in *k*_obs_ was also registered.

The extension of the RNA–DNA hybrid part (R-loops 6–8) affected RNA primer cleavage in different ways. For R-loop 6 (with a 16-nt RNA–DNA hybrid part), a lower overall extent of primer cleavage but not a reduced cleavage rate was observed. A similar picture was seen with R-loops 7 and 9. It is worth mentioning that, in the case of R-loop 8, an increase in both the overall extent of primer cleavage and *k*_obs_ was observed (Figure 6).

## 3. Discussion

**Figure 7:**
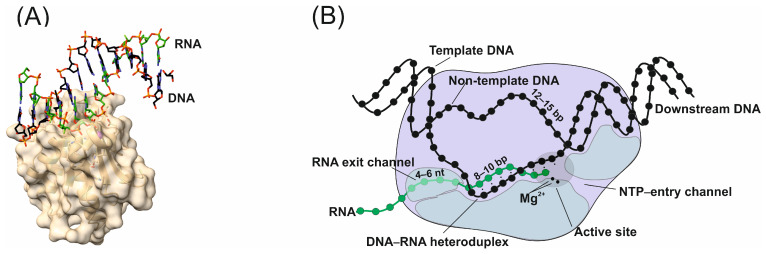
(**A**) The crystal structure of a complex of the catalytic domain of *Bacillus halodurans* RNase HI with RNA–DNA hybrids (PDB ID 5SWM) [68]. (**B**) The structural features of the transcription elongation complex.

RH1 is an enzyme possessing a mixed α/β structure and containing a carboxylate triad in the catalytic site [64]. The RH1 molecule is composed of two subdomains. The principal domain contains four α helices and one large sheet at the N terminus, and the minor domain consists of one α helix and a following loop containing basic residues. It is believed that a DNA–RNA hybrid double helix binds to the basic minor domain [64]. Stoichiometric analyses of RH1 binding to substrates suggest that RH1 binds to 9–10 bp of the RNA–DNA hybrid [65]. It has been found that DNA bases complementary to the RNA bases located six or seven nucleotides upstream of the cleavage site interact with the enzyme basic protrusion, regardless of whether it is hybridized to the RNA strand or not [66]. For productive binding, the axis from the 3′ to the 5′ end of the RNA strand of the substrate duplex must be oriented in agreement with the vector from the active site to the enzyme basic protrusion [67]. In BDB, there is a crystal structure of a complex of the catalytic domain of *Bacillus halodurans* RNase HI (BhRNase H) [68] with RNA–DNA hybrids (Figure 7A). It was shown that RNase H specifically recognizes the A conformations of the RNA strand and the B-form conformation of the DNA strand. RNase H interacts with the minor groove of the heteroduplex. The interaction of five consecutive 2′-hydroxyl groups of the ribose residues with four amino acids of RNase H enables the enzyme to distinguish between RNA and DNA chains [68]. The two metal ions are asymmetrically coordinated and have different roles in activating the nucleophile and stabilizing the transition state. The metal ion A produces the hydroxy anion nucleophile that attacks the scissile phosphate group, and metal ion B participates in leaving group stabilization. The enzyme bridges RNA and DNA across the minor groove through the phosphate group of the DNA strand 2 bp from the scissile phosphate [69].

In our work, we used a set of model R-loops imitating a transcription elongation complex. The strands forming R-loops in vivo are the parts of the coding DNA region and are complementary. RNA polymerase binds with the 3′ end of the transcribed DNA region and then slides along the DNA, melting the DNA and synthesizing an mRNA chain. The nascent RNA comes out of the RNA exit channel, and its rehybridization with the DNA template strand is typically prevented by spatial separation, resulting in the formation of the DNA double-stranded structure after the transcription elongation complex. However, under certain conditions, the nascent RNA can bind to the DNA template behind the transcribing RNAP, resulting in the formation of an R-loop structure containing a long and stable RNA–DNA hybrid and a displaced ssDNA. Using model R-loop structures allowed us to determine the kinetic features of the interaction of RH1 with R-loops of various structures and lengths in the presence and absence of RNAP. The dissociation constants *K*_d_ determined using MST for RH1 binding to R-loops were 1.4 ± 0.3 for R-loops 1–4 and 7.7 ± 2.2 for R-loops 6–9. R-loops 1–5 contained a 10–11-nt RNA–DNA hybrid part, whereas in R-loops 6–9, the RNA–DNA hybrid part was varied from 16 to 32 nt. An interesting finding was obtained for R-loop 5; it had a 3.8-fold-lower *K*_d_ compared to R-loop 1, possibly indicating an optimal conformation of the RNA–DNA hybrid part in R-loop 5 for productive binding and subsequent catalysis. Our kinetic data on RNA primer cleavage by RH1 are in good agreement with the MST data, suggesting that the rate-limiting step of the process is catalytic-complex formation.

To reconstruct the molecular interactions between participants in a transcription–replication collision in vitro, the RH1-driven cleavage of an RNA primer contained in the R-loops in the presence of the RNAP was investigated. The transcription elongation complex (Figure 7B) includes a downstream entry channel for DNA, a melted transcription bubble of 12–15 bp with an 8–10 bp DNA–RNA heteroduplex, an RNA exit channel that holds 4–6 nucleotides of single-stranded RNA, a secondary entry channel for NTP, and the active site in which NTPs react with the 3′-OH on RNA to extend the RNA strand. The repeat hybridization of RNA with the DNA template strand is prevented by the spatial displacement of these strands from each other, leading to the formation of the DNA double-stranded structure after the translocation of the transcription elongation complex [60]. Examination of the cleavage of the RNA primer contained in the R-loops by RH1 in the presence of the RNAP pointed to a correlation between the length of the RNA–DNA hybrid part and the efficacy of RNA primer digestion by RH1. For R-loops 1–6, a lower overall extent of primer cleavage was noted. R-loops 1–5 contain a 10–11-nt RNA–DNA hybrid part, which is “typical” in a transcription elongation complex. The RNAP covers the surface of the hybrid part, thereby restricting the access of RH1 to the RNA primer. Taking into consideration the *K*_d_ value for the complex of the RNAP with R-loop 1, 0.3 µM, the maximal proportion of the [RNAP•R-loop] complex is 68%. As readers can see in Figure 6, the overall extent of primer cleavage varied from 40% to 60%, implying almost full protection of the RNA primer in the [RNAP•R-loop] complex from RH1 action. For R-loop 6 (containing a 16-nt RNA–DNA hybrid part), such protection probably is caused by small “landing place” for RH1 remaining after RNAP binding. For R-loops 7–9, there was no protection of the RNA primer by the RNAP because the size of the “landing site” remaining after RNAP binding was sufficient for RH1. As a consequence, within the R-loop, RH1 could cleave both the hybrid duplex’s “free” segment (which is unbound by the RNAP) and the RNA–DNA hybrid part’s 3′ end (which is bound by the RNAP). The degradation of the 5′ end of the RNA primer and an inability of the RNAP to move forward due to NTP absence caused RNAP dissociation and full degradation of the RNA primer by RH1.

## 4. Materials and Methods

### 4.1. Enzymes and Oligonucleotides

*E. coli* strain Arctic (DE3) carrying the wild-type version of the *E. coli* RH1 gene with an N-terminal 6×His tag in the pET28c vector (Novagen, Merck KGaA, Darmstadt, Germany) was grown on Luria broth with 50 µg/ml kanamycin until the A_600_ reached 0.6, after which 0.2 mM IPTG was added, and the incubation was continued for 3 h at 37 °C. All the subsequent procedures were performed at 4 °C. Cell pellets from a 300 mL culture were resuspended in 3 mL of a buffer composed of 20 mM HEPES-NaOH, pH 7.8, 40 mM NaCl, and a mixture of protease inhibitors (Inhibitor cocktail, Complete, Mannheim, Germany) and were disrupted through ultrasonication (10 repeats of a 10 s pulse at a frequency of 20 kHz followed by a 30 s pause). The resultant cell lysate was centrifuged (40 min at 40,000× *g*); a solution of NaCl and imidazole was added to the supernatant to concentrations of 500 and 30 mM, respectively; and the mixture was applied to a Polar MC30-Ni column (Sepax Technologies, Inc., Newark, DE, USA) at a flow rate of 0.4 mL/min. The chromatography was performed in a buffer consisting of 20 mM HEPES-NaOH, 500 mM NaCl, and a linear 30-to-600 mM gradient of imidazole; the optical density of the solution at 280 nm was recorded. The obtained enzyme-containing fraction was diluted to a final NaCl concentration of 50 mM and applied to a HiTrap-Heparin™ column (Amersham Biosciences, Uppsala, Sweden) at a flow rate of 0.4 mL/min. The chromatography was performed in a buffer composed of 20 mM HEPES-NaOH and a linear gradient of NaCl (50 to 600 mM), with the detection of the optical density at 280 nm. The purity of the RH1 was determined using gel electrophoresis. A catalytically inactive mutant—RH1 D10N [63]—was generated by site-directed mutagenesis using the oligonucleotides RH1 D10N_F 5′-GCTTAAACAGGTAGAAATTTTCACCAATGGTTCGTGTCTGGGCAATCC-3′ and RH1 D10N_R 5′-GGATTGCCCAGACACGAACCATTGGTGAAAATTTCTACCTGTTTAAGC-3′ and confirmed through sequencing. RH1 D10N was expressed and purified using the same procedure as for wild-type RH1. A recombinant *E. coli* RNAP was prepared and purified as described previously [70].

### 4.2. Preparation of R-Loops

DNA oligonucleotides were acquired from Biosset Ltd. (Novosibirsk, Russia). RNA oligonucleotides with FAM at the 5′ end were synthesized in the Laboratory of RNA Chemistry at ICBFM SB RAS (Novosibirsk, Russia) as described before [71]. Table 1 contains the sequences of the model R-loops. The concentrations of oligonucleotides were calculated from their absorbance at 260 nm. R-loops were prepared via the annealing of nontemplate and template DNA strands and RNA primer at a 1:1:1 molar ratio in H_2_O. R-loop 9 was prepared via the annealing of nontemplate and template DNA strands and an RNA primer at a 1:1:1 molar ratio in a buffer composed of 10 mM Na_2_HPO_4_, 1.8 mM NaH_2_PO_4_ (pH 7.5), 1 mM EDTA, 1 mM DTT, and 140 mM KCl. The R-loop formation was characterized using the electrophoretic mobility shift assay (EMSA) in a nondenaturing 10% polyacrylamide gel (75:1) in 0.5× TBE buffer + 2 mM MgCl_2_ (for R-loop 1–7) and +5 mM MgCl_2_ (for R-loop 8, 9) at 10 mA and 4 °C. The prepared annealed solutions of 10x R-loop were stored at –20 °C. In all cases, unless otherwise stated, the R-loops were prepared using this method.

### 4.3. R-Loop-Binding Assays

The stability constants of the complexes formed between each R-loop and the catalytically inactive mutant of RH1 (D10N) were determined using a Monolith NT.115 system (NanoTemper Technologies) using standard capillaries (Monolith NT.115 Standard Treated Capillaries, NanoTemper Technologies GmbH, Munich, Germany). Each data point of the titration curves was obtained by measuring the fluorescence intensity of individual solutions (10 μL) composed of an R-loop (0.5 μM) and the enzyme (0.05–30.00 μM) in a buffer (40 mM Tris-HCl pH 7.9, 40 mM KCl, and 10 mM MgCl_2_) at 25 °C. To calculate the dissociation constants, the experimental data were processed in the OriginPro 8.1 SR3 software v8.1.34.90 (OriginLab Corporation, Northampton, MA, USA) under the single-stage-binding model.

### 4.4. RNase H Activity Assay

To evaluate the activity of RH1 on different R-loops, a solution of an R-loop (1 µM) was mixed with an enzyme solution (20–200 nM) at 25 °C in a buffer consisting of 40 mM Tris-HCl pH 7.9, 40 mM KCl, and 10 mM MgCl_2_. At certain time intervals, an aliquot (10 µL) was taken, and an equal volume of a stop solution (9 M urea, 50 mM EDTA, 0.1% of xylene cyanole, and 0.1% of bromophenol blue) was introduced. The reaction products were separated in a denaturing 15% polyacrylamide gel. The resulting gel was visualized using a VersaDoc gel-documenting system (Bio-Rad Laboratories, Hercules, CA, USA). The degree of substrate transformation was computed as the ratio of the peak areas of the product to the sum of the peak areas of the product and of the peak of the initial substrate, using the GelAnalyzer 23.1.1 software (©Istvan Lazar Jr.). The obtained data were fitted to the equation [Product] = A × (1 − exp(−*k*_obs_ × *t*)), where A is the amplitude, *k*_obs_ is the rate constant, and *t* is the reaction time.

### 4.5. RNase H Activity Assay in the Presence of RNAP

A pre-formed [RNAP•R-loop] complex was assembled according to the following procedure. A heteroduplex consisting of a template strand (1.1 µM) and an RNA primer (1 µM) was mixed with the RNAP (2 µM) in a buffer consisting of 40 mM Tris-HCl, pH 7.9, 40 mM KCl, and 10 mM MgCl_2_ and kept on ice for 10 min; then, a nontemplate strand (2 µM) was added, and the solution was incubated on ice for an additional 20 min. After that, a solution of the pre-formed [RNAP•R-loop] complex was incubated for 1 min at 25 °C and mixed with an equal volume of an RH1 solution (50 nM). At certain time intervals, an aliquot (10 µl) was taken, and an equal volume of the stop solution was introduced. The reaction products were separated in a denaturing 15% polyacrylamide gel and analyzed in the same manner as for the RNase H activity assay in the absence of the RNAP.

## 5. Conclusions

In this study, the kinetic features of the interaction of RH1 with R-loops of various structures were investigated. The binding of an R-loop by RH1 was more effective for R-loops containing a 10–11-nt RNA–DNA hybrid part. The kinetic data for RNA primer cleavage by RH1 indicate that the rate-limiting step of the process is catalytic-complex formation. In the presence of RNAP, our findings on the cleavage of the RNA primer also indicate a correlation between the length of the RNA–DNA hybrid part and the efficacy of RNA primer digestion by RH1. For R-loops containing a ≤16-nt RNA–DNA hybrid part, almost full protection of the RNA primer from RH1 action was observed in the [RNAP•R-loop] complex. A longer RNA–DNA hybrid part was cleaved effectively by RH1.

## Figures and Tables

**Table 1:** Sequences and structures of model R-loops.

Shorthand	Nucleotide Sequence
R-loop 1	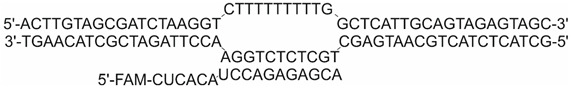
R-loop 2	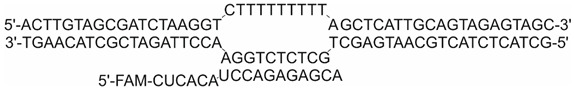
R-loop 3	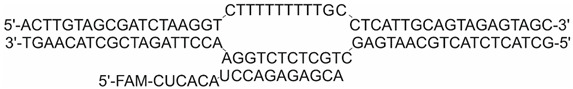
R-loop 4	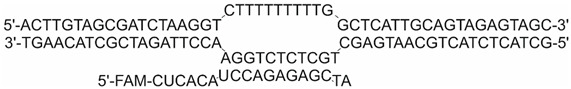
R-loop 5	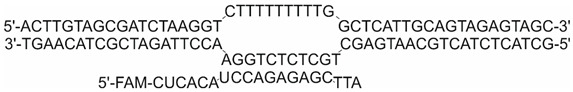
R-loop 6	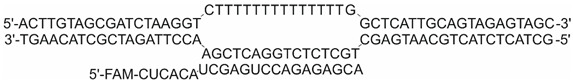
R-loop 7	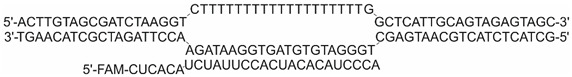
R-loop 8	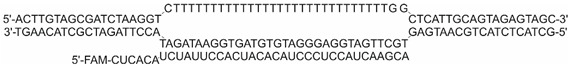
R-loop 9	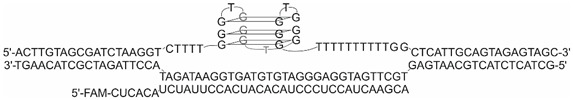

**Table 2:** Dissociation constants *K*_d_ for R-loop binding by RH1 D10N, and the rate constants obtained from the cleavage data.

Substrate	*K*_d_, µM	*k*_1_, M^−1^⋅s^−1^, ×10^−5^
R-loop 1	1.2 ± 0.4	28 ± 3
R-loop 2	1.1 ± 0.4	38 ± 8
R-loop 3	1.5 ± 0.4	36 ± 2
R-loop 4	1.8 ± 0.4	27 ± 2
R-loop 5	0.35 ± 0.15	65 ± 5
R-loop 6	9.7 ± 2.7	14 ± 3
R-loop 7	6.7 ± 3.1	16 ± 2
R-loop 8	9.3 ± 4.8	5.8 ± 0.5
R-loop 9	5.1 ± 1.4	9.1 ± 0.9

## Data Availability

The original contributions presented in the study are included in the article; further inquiries can be directed to the corresponding authors.
